# Comparison of Serological Detection Methods for *Toxoplasma gondii* Antibodies and Seroprevalence in Captive Red Pandas

**DOI:** 10.3390/ani16030396

**Published:** 2026-01-27

**Authors:** Chanjuan Yue, Wanjing Yang, Dunwu Qi, Yanshan Zhou, Xueyang Fan, Chao Chen, Yifan Wen, Xiaolan Wang, Mei Yang, Yunli Li, Rong Hou, Songrui Liu

**Affiliations:** Chengdu Research Base of Giant Panda Breeding, The Conservation of Endangered Wildlife Key Laboratory of Sichuan Province, 1375 Panda Road, Chenghua District, Chengdu 610081, China

**Keywords:** modified agglutination test (MAT), enzyme-linked immunosorbent assay (ELISA), toxoplasmosis, serological test, ex-situ conservation

## Abstract

*Toxoplasma gondii* is a globally distributed zoonotic parasite that infects a wide range of warm-blooded animals and poses serious threats to many endangered species. Effective serological detection is critical for disease surveillance and control, yet the accuracy of commercial assays varies significantly across host species. Validated serological methods for the Chinese red panda (*Ailurus styani*) remain scarce, limiting effective monitoring and conservation efforts. A total of 57 serum samples were collected from captive red pandas. Three commercial serological kits—the modified agglutination test (MAT), indirect hemagglutination assay (IHA), and enzyme-linked immunosorbent assay (ELISA)—were used to detect *T. gondii* antibodies. Statistical analyses included agreement assessment (Cohen’s kappa), McNemar’s test, and receiver operating characteristic (ROC) curve analysis, with MAT as the reference standard. MAT detected *T. gondii* antibodies in 36.84% (21/57; 95% CI: 24.32–49.36) of samples, while ELISA identified 42.11% (24/57; 95% CI: 29.29–54.92). IHA failed to detect any positive samples (0%). MAT and ELISA showed almost perfect agreement (κ = 0.817; 95% CI: 0.66–0.97) with no significant difference (*p* > 0.05). Compared to MAT, ELISA exhibited 95.2% sensitivity and 88.9% specificity. The combined MAT/ELISA approach with retesting of discordant samples yielded a final seroprevalence of 35.09% (20/57), with 8.77% (5/57) indeterminate results. MAT and ELISA are reliable methods for serological surveillance of *T. gondii* in red pandas, while IHA is unsuitable. We recommend MAT for initial screening followed by ELISA confirmation. The high seroprevalence highlights the need for integrated control strategies, including management of stray felids and rodents in captive environments to reduce transmission risks.

## Simple Summary

*Toxoplasma gondii* is a globally distributed parasite that threatens many warm-blooded species, including endangered wildlife such as the red panda. Reliable detection of exposure is essential for monitoring health in captive populations, but the performance of commercial antibody tests can vary across species. This study evaluated the modified agglutination test (MAT), indirect hemagglutination assay (IHA), and enzyme-linked immunosorbent assay (ELISA) for detecting *T. gondii* antibodies in 57 serum samples from captive red pandas. Results showed that MAT and ELISA had almost perfect agreement and were both effective, whereas IHA failed to detect any positive samples. The combined use of MAT and ELISA determined a seroprevalence of 35.09%, indicating a substantial exposure risk in captivity. These results validate MAT and ELISA as reliable tools for *T. gondii* surveillance in red pandas and underscore the importance of managing potential transmission sources, such as stray cats and rodents, in captive settings. This work supports evidence-based health monitoring and targeted disease prevention for red panda conservation.

## 1. Introduction

The Chinese red panda (*Ailurus styani*; hereafter referred to as red panda) is an endemic species protected under China’s Class II designation and classified as Endangered by the International Union for Conservation of Nature (IUCN) [1]. Its population has declined by approximately 40% over the past 50 years, primarily due to habitat destruction and fragmentation [2]. Emerging infectious diseases, particularly parasitic infections, now pose significant threats to both in situ and ex situ populations. Over 20 parasite species have been documented in red pandas [3]. Among these, toxoplasmosis—a globally distributed parasitic disease caused by *Toxoplasma gondii*—represents a parasitic disease of substantial concern for red panda conservation.

*Toxoplasma gondii* (*T. gondii*) infects most warm-blooded animals, including red pandas [4]. Infections may cause reproductive failure or acute toxoplasmosis in immunocompromised hosts. Previous studies have reported cases of wildlife mortality attributable to *T. gondii* infection globally [5,6].

Serological assays are the primary method for detecting *T. gondii* exposure in wildlife and captive animals [7]. Previous studies have employed the modified agglutination test (MAT), indirect hemagglutination assay (IHA), enzyme-linked immunosorbent assay (ELISA), and immunofluorescence antibody test (IFAT) [8,9,10,11], though the accuracy of these methods varies. MAT and ELISA are widely used in epidemiological studies due to their high sensitivity and applicability across species [4]. Although concurrent MAT and ELISA testing has been reported in captive wildlife, including four red pandas [10], small sample sizes limit robust method comparisons. Therefore, validating reliable serological methods for red pandas remains necessary. To enhance health monitoring for captive red panda conservation, this study aimed to evaluate and compare three commercial serological kits (MAT, IHA, and ELISA) for detecting *T. gondii* antibodies in captive red pandas in order to identify reliable serosurveillance tools and to determine the seroprevalence in a captive population.

In this study, fifty-seven blood samples were collected from captive red panda individuals in Chengdu, China. The performance of two commercial serological kits (IHA and ELISA) was evaluated against the MAT reference standard for detecting *T. gondii* antibodies. MAT was adopted as the reference standard for comparison because it is not species-specific and is widely established as a benchmark for evaluating *T. gondii* antibody detection in wildlife serology. Analytical performance, including sensitivity and specificity, was assessed for each commercial kit relative to MAT. Based on comparative results, optimal serological methods for *T. gondii* surveillance in red pandas were identified, and the seroprevalence in this captive population is reported.

## 2. Materials and Methods

### 2.1. Sample Collection

Fifty-seven blood samples were collected from the cephalic vein of red pandas at the Chengdu Research Base of Giant Panda Breeding (CRBGPB) in Sichuan, China, between November 2015 and July 2019. All blood samples were collected opportunistically during routine health examinations. At the time of sampling, the animals displayed no overt clinical signs suggestive of active parasitic or other infectious diseases. The animal handling procedures were approved by the Institutional Animal Care and Use Committee (IACUC) of the Chengdu Research Base of Giant Panda Breeding (NO. 2019013; Approval Date: 21 November 2019). Serum was separated by centrifugation at 3500 rpm for 10 min and stored at −80 °C.

### 2.2. Serological Testing

All samples were tested using three commercial kits with three detection methods (Table 1). The MAT kit had a cut-off titer of 1:25, and samples were diluted in twofold serial dilutions from 1:25 to 1:200. The IHA kit had a cut-off titer of 1: 4, and samples were diluted in twofold serial dilutions from 1: 4 to 1: 256. The optical density (OD) values obtained from the ELISA kit were measured using a Multiskan^TM^ Microplate Absorbance Reader (Thermo Scientific, Singapore). All experimental procedures were performed according to the manufacturer’s instructions.

### 2.3. Seroprevalence Determination

Based on comparative performance results, we selected appropriate commercial kits for *T. gondii* antibody detection in red panda serum. A dual-method approach was implemented to determine seroprevalence, in which samples were classified as positive only when both methods yielded positive results. Samples with discordant results were retested using the same serum aliquot. Samples exhibiting persistent discordance after retesting were classified as negative.

### 2.4. Statistical Analysis

All statistical analyses were performed using R version 4.5.0 (R Core Team, 2025) with the following packages: tidyverse (v2.0.0), irr (v0.84.1), pROC (v1.19.0.1), and binom (v1.1.1.1).

Seroprevalence rates with 95% confidence intervals were calculated using the Wilson score method. The McNemar chi-square test with continuity correction was used to compare detection rates between MAT and ELISA (*p* < 0.05 considered significant). Agreement between methods was assessed using Cohen’s kappa coefficient (κ) with the following interpretations: κ ≤ 0.20 = slight agreement; 0.21–0.40 = fair agreement; 0.41–0.60 = moderate agreement; 0.61–0.80 = substantial agreement; and 0.81–1.00 = almost perfect agreement.

Using MAT as the reference standard, the sensitivity, specificity, positive predictive value (PPV), negative predictive value (NPV), and accuracy of ELISA were calculated. The area under the curve (AUC) was evaluated by receiver operating characteristic (ROC) analysis using the continuous ELISA optical density values. The ROC curve analysis was performed using the raw optical density (OD) values obtained from the ELISA, as the kit manufacturer’s protocol specifies direct use of the measured OD values without conversion.

## 3. Results

### 3.1. Detection Rates

According to manufacturer-specified cut-offs, the MAT detected *T. gondii* antibodies in 21 of 57 red pandas (36.84%; 95% CI: 25.50–49.80), with titers distributed as follows: 1: 50 (*n* = 2) and 1: 200 (*n* = 19) (Table 2). The ELISA identified 24 seropositive samples (42.11%; 95% CI: 30.2–55.0). In contrast, the IHA detected no seropositive results (0%) (Table 3).

MAT served as the reference standard for serological comparisons. Among 57 red panda serum samples, IHA yielded no positive results (Table 3), resulting in 100% false negatives (21/21 MAT-positive samples undetected) and 0% false positives. ELISA demonstrated one false-negative result (negative by ELISA but positive by MAT; 4.76% [1/21] of MAT-positive samples) and four false positives (11.11% [4/36] of MAT-negative samples).

### 3.2. Agreement Analysis

The commercial IHA kit failed to detect *T. gondii* antibodies in any red panda serum samples and was therefore excluded from further analysis. Cohen’s kappa coefficient (κ) analysis revealed almost perfect agreement between MAT and ELISA for detecting *T. gondii* exposure in red pandas (κ = 0.817, 95% CI: 0.66–0.97). McNemar’s test demonstrated no significant difference between methods (*p* = 0.375, χ^2^ = 0.800).

### 3.3. ROC Analysis

**Figure 1:**
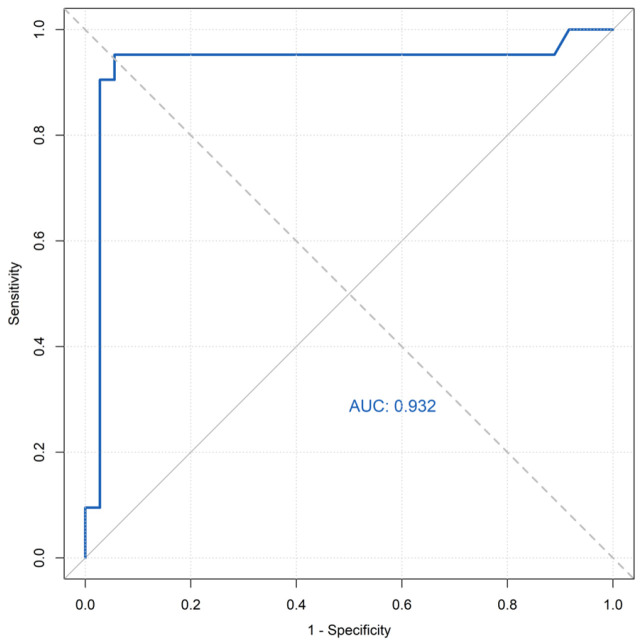
ROC curve for ELISA detection of *T. gondii* antibodies in red pandas using MAT as reference standard.

Using MAT as the reference standard, ROC analysis of ELISA yielded an area under the curve (AUC) of 0.932 (95% CI: 0.837–1.000), indicating high diagnostic accuracy (Figure 1). Compared to MAT, ELISA demonstrated 95.20% sensitivity and 88.9% specificity.

### 3.4. Seroprevalence of T. gondii in Captive Red Pandas

Using a combined MAT/ELISA approach with retesting of discordant samples, the seroprevalence of *T. gondii* in captive red pandas was determined to be 35.09% (20/57). Five samples (8.77%) remained discordant after retesting and were classified as negative.

## 4. Discussion

The red panda has been identified as an intermediate host for *T. gondii* [12]. A fatal case of disseminated toxoplasmosis in this species was documented by Stief et al. (2012) [13], with histopathological examination revealing abundant tissue cysts in the liver and brain, and fewer cysts in other organs. Additionally, Ashley et al. (2020) [14] demonstrated disseminated toxoplasmosis in four red pandas during necropsy through PCR, IHC, and MAT. Given that most wildlife infections remain subclinical [4], serological surveillance provides significant advantages for investigating toxoplasmosis in wildlife due to its non-lethal sampling and operational efficiency.

Serological methods including the MAT, IHA, and ELISA constitute primary diagnostic tools for detecting *T. gondii* exposure in wildlife [4]. MAT demonstrates particular utility in wildlife serosurveillance due to its simple protocol, high sensitivity, broad species applicability, and minimal equipment requirements [15]. However, interpretive subjectivity necessitates combining MAT with complementary serological methods to enhance detection accuracy [7]. While ELISA requires photometric equipment for result interpretation, its commercial availability, procedural simplicity, and objective output facilitate standardized testing [16].

Our methodological validation demonstrates nearly perfect agreement (κ = 0.817) between MAT and ELISA for *T. gondii* antibody detection in red pandas, consistent with findings in giant pandas and other wildlife [9,12]. ELISA exhibited high diagnostic performance relative to MAT (sensitivity: 95.2%; specificity: 88.9%; AUC: 0.932), supporting its reliability for serosurveillance. In contrast, IHA failed to detect any seropositive samples, demonstrating complete inadequacy for red panda testing. This aligns with known limitations of IHA, including poor repeatability and reduced sensitivity compared to MAT [13,14]. The observed MAT titer distribution, with a predominance of the 1:200 titer, may reflect a relatively homogeneous exposure intensity or timing within this captive population, though individual immunological variation could also be a factor.

In this study, MAT served as the reference standard for serological comparisons. No significant difference (*p* > 0.05) was observed between ELISA and MAT for detecting *T. gondii* antibodies in captive red pandas. Relative to MAT, ELISA demonstrated 95.2% sensitivity and 88.9% specificity, with ROC analysis confirming strong diagnostic agreement (AUC = 0.932). These findings align with previous MAT-ELISA concordance reports in captive giant pandas and other wildlife [10,17].

Furthermore, IHA demonstrates substantial limitations for red panda serosurveillance despite its utility in livestock screening. While MAT and ELISA consistently detected *T. gondii* antibodies in 35.09% (20/57) of captive red pandas, IHA failed to identify any seropositive samples. This detection failure aligns with documented methodological constraints of IHA, including poor repeatability and erythrocyte instability [18]. Supporting evidence emerges from prior studies. Qin et al. (2007) [9] reported only 19.2% seroprevalence using IHA in 73 red pandas (including CRBGPB seronegatives), while Loeffler et al. (2007) [19] detected zero seropositivity in CRBGPB specimens. These findings collectively validate IHA’s reduced sensitivity relative to MAT [20] and confirm its inadequate performance in wildlife serosurveillance [10].

The seroprevalence of *T. gondii* antibodies observed in this study (35.09%, 20/57) markedly exceeds rates reported in prior investigations of red pandas, a discrepancy primarily attributable to methodological variations (Table 4). Qin et al. (2007) [9] employed IHA—a method with documented sensitivity limitations—and reported only 19.2% seroprevalence. In contrast, studies using MAT or ELISA consistently yield higher detection rates. Chen et al. (2015) [8] applied MAT to captive red pandas at Fuzhou Zoo, identifying 21.05% seropositivity (4/19), while Yang et al. (2019) [12] detected 12.5% (1/8) in pneumonic individuals using the same method. Zhang et al. (2000) [10] reported 75% seroprevalence (3/4) in Shanghai Zoo red pandas using combined MAT/ELISA, though this small sample size limits generalizability. Notably, captive giant pandas housed under identical environmental conditions at CRBGPB exhibited nearly identical seroprevalence (35.67%, 56/157) when tested via MAT/ELISA [21].

Acquired infection may represent the primary route of *T. gondii* transmission. Felids serve as definitive hosts for *T. gondii*, with environmental contamination from oocyst-shedding feces constituting the principal transmission pathway [4]. Although red pandas cohabitate with giant pandas, their broader dietary ecology increases infection risk. In addition to consuming bamboo and prepared foods, captive red pandas engage in opportunistic predation on *T. gondii*-infected intermediate hosts (rodents and birds). This behavior substantially elevates exposure through tissue cyst ingestion. Consequently, implementing targeted biosecurity measures against stray felids and reservoir rodents is critical for mitigating transmission in captive management programs.

## 5. Conclusions

Based on the comparative analysis of serological methods for detecting *Toxoplasma gondii* antibodies in captive red pandas, it is concluded that both the modified agglutination test (MAT) and enzyme-linked immunosorbent assay (ELISA) demonstrate high reliability and strong agreement (κ = 0.817), with no significant difference in detection rates. ELISA showed high sensitivity (95.2%) and specificity (88.9%) relative to MAT. In contrast, the indirect hemagglutination assay (IHA) proved entirely unsuitable, failing to detect any positive samples. The final seroprevalence was determined to be 35.09% using a combined MAT/ELISA approach with retesting of discordant results. The high concordance between MAT and ELISA for detecting *T. gondii* antibodies in red pandas supports a combined strategy of MAT screening followed by ELISA confirmation in serosurveillance programs. The high seroprevalence underscores the need for integrated management strategies to reduce transmission risks, including control of stray felids and rodents in captive environments.

## Figures and Tables

**Table 1:** The summary information of the three commercial kits.

Test	Name	Kit Source	Link	Host Species	Cut-Off/Criterion	
MAT	*Toxoplasma* Modified Agglutination Test (MAT) Kit	University of Tennessee Research Foundation, Technology Transfer & Licensing, Memphis, TN, USA	https://utrf.tennessee.edu/product/tgmat-reference-dna/ (accessed on 5 March 2025)	multiple species	Titer ≥ 1:25	
IHA	The Indirect Hemagglutination Assay Kit for Detection of Antibodies Against *Toxoplasma gondii*	Lanzhou Shouyan Biotechnology Co., Ltd., Lanzhou, China	https://datt.caas.cn/zdtgcg/xcp/cmsyl/61321.htm (accessed on 15 May 2025)	multiple species	Titer ≥ 1:4	
ELISA	Diagnostic Kit for IgG Antibody to *Toxoplasma* (ELISA)	Haitai Biological Pharmaceuticals Co., Ltd., Zhuhai, China	http://zhhaitai.com/product.asp?id=194 (accessed on 23 January 2025)	multiple species	Positive	OD_S_ > OD_C_ × 1.1
					Doubtful	OD_C_ × 0.9 ≤ OD_S_ ≤OD_C_ × 1.1
					Negative	OD_S_ < OD_C_ × 0.9

OD_S_: optical density of sample; ODc: optical density of control sample.

**Table 2:** Antibody titers to *T. gondii* measured by the MAT test.

MAT Titer					Total
<1: 25	1: 25	1: 50	1: 100	1: 200	
36	0	2	0	19	57

**Table 3:** Cross-classification of results and test performance.

	MAT	IHA		ELISA	
		+	−	+	−
Cross-classification	+	0	21	20	1
	−	0	36	4	32
False-negative rate (%)		0		4.76	
False-positive rate (%)		100		11.11	
Seroprevalence (%)	36.84	0		42.11	
95% CI	25.5–49.8	0		30.2–55.0	

Calculations assume MAT as reference. False-negative rate = (MAT+/IHA−)/(MAT+). False-positive rate = (MAT−/IHA+)/(MAT−).

**Table 4:** Investigations of *T. gondii* IgG antibody seroprevalence in red pandas of previous studies.

Species	Location	Method	Seroprevalence	Sample Size	Reference
Red Panda	Chengdu (This Study)	MAT/ELISA	35.09%	57	Current Data
Red Panda	Multiple Sites	IHA	19.2%	73	[9]
Red Panda	Fuzhou Zoo	MAT	21.05%	19	[8]
Giant Panda	CRBGPB	MAT/ELISA	35.67%	157	[21]

## Data Availability

The data used in this article are available on request by contacting the corresponding author.
